# Disclosure of cancer diagnosis and quality of life in cancer patients: should it be the same everywhere?

**DOI:** 10.1186/1471-2407-9-39

**Published:** 2009-01-29

**Authors:** Ali Montazeri, Azadeh Tavoli, Mohammad Ali Mohagheghi, Rasool Roshan, Zahra Tavoli

**Affiliations:** 1 Iranian Institute for Health Sciences Research, ACECR, Tehran, Iran; 2 Department of Psychology, Faculty of Humanity Studies, Shahed University, Tehran, Iran; 3 Cancer Research Centre, Cancer Institute, Tehran, Iran

## Abstract

**Background:**

Evidence suggests that truth telling and honest disclosure of cancer diagnosis could lead to improved outcomes in cancer patients. To examine such findings in Iran, this trial aimed to study the various dimensions of quality of life in patients with gastrointestinal cancer and to compare these variables among those who knew their diagnosis and those who did not.

**Methods:**

A consecutive sample of patients with gastrointestinal cancer being treated in Cancer Institute in Tehran, Iran was prospectively evaluated. A psychologist interviewed patients using the Iranian version of the European Organization for Research and Treatment of Cancer Quality of Life Questionnaire (EORTC QLQ-C30). Patients were categorized into two groups: those who knew their diagnosis and those who did not. Independent sample t-test was used for group comparisons.

**Results:**

In all 142 patients were interviewed. A significant proportion (52%) of patients did not know their cancer diagnosis and 48% of patients were aware that they had cancer. They were quite similar in most characteristics. The comparison of quality of life between two groups indicated that those knew their diagnosis showed a significant lower degree of physical (P = 0.001), emotional (P = 0.01) and social functioning (P < 0.001), whereas the global quality of life and other functional scales including role functioning and cognitive functioning did not show significant result. There were no statistically significant differences between symptoms scores between two groups, except for fatigue suggesting a higher score in patients who knew their diagnosis (P = 0.01). The financial difficulties were also significantly higher in patients who knew their cancer diagnosis (P = 0.005). Performing analysis of variance while controlling for age, educational status, cancer site, and knowledge of cancer diagnosis, the results showed that the knowledge of cancer diagnosis independently still contributed to the significant differences observed between two groups.

**Conclusion:**

Contrary to expectation the findings indicated that patients who did not know their cancer diagnosis had a better physical, social and emotional quality of life. It seems that due to cultural differences between countries cancer disclosure guidelines perhaps should be differing.

## Background

A diagnosis of cancer often imposes a crisis, with the person having to confront the illness and its treatment, and deal with issues concerning the meaning of life, death, and an uncertain future [1]. Thus historically there was a belief that a patient should not be told about his or her cancer diagnosis, and the question 'should patients be told they have cancer?' has been addressed for a long time as one of the most studied aspects in the literature of cancer communication [2]. While many argue that it is important to give patients this information so that they can make important decisions in an informed manner, others suggest that giving this sort of information can destroy hope. Moreover, based on some broad cultural differences among those who hold one of these views, whether or not to tell the truth about diagnoses and prognoses in such situations has arguably come to symbolize the importance of cross-cultural differences in medical practice [3,4].

Cancer and its treatments often produce significant morbidities that undermine quality of life in survivors [5]. Quality of life (QOL) assessment is now considered as an important component of evaluation in chronic disease, particularly in cancer clinical trials [6]. There are few studies that investigate quality of life in cancer patients regarding knowledge of cancer diagnosis. According to a study from India, psychiatric morbidity is significantly lower in patients who are "unaware" of the diagnosis of cancer and who have a more hopeful outlook of the treatment [7]. A study performed in Turkey, indicated that psychiatric morbidity was significantly higher in patients who knew that they had a cancer diagnosis. These findings suggest that the awareness of cancer diagnosis is related to the presence of psychiatric morbidity [8]. On the contrary, some studies have shown that honest disclosure of the truth does not worsen any dimension of quality of life in general or emotional functioning in particular [9,10].

Since no studies has investigated the quality of life of Iranian patients regarding knowledge of cancer diagnosis, here we investigated about quality of life of Iranian cancer patients; and compared it among those who knew their diagnosis and those who did not. Because of high incidence of esophageal and stomach cancer in Iran [11] it was decided to select gastrointestinal cancer patients in an attempt (i) to examine quality of life in these group of cancer patients and (ii) to compare these variables among those who knew their diagnosis and those who did not.

## Methods

### Design and data collection

An interview based prospective study was carried out to measure quality of life in patients with gastrointestinal cancer. Data were collected during November 2005 and April 2006. The intention was to interview all gastrointestinal cancer inpatients attending to a large teaching hospital (Imam Hospital) for their treatment in Tehran, Iran. To assess patient's knowledge of the cancer diagnosis, both patients and relatives were investigated separately. This was achieved at the end of each interview. First we asked relatives to indicate whether a patient knew his or her diagnosis. Then to confirm this with patients, after a careful consideration each patient was asked what was wrong with he or she. Knowledge was assessed by patient's ability to acknowledge the illness and use the terms 'cancer' or 'tumor'. In Iran usually educated people use 'cancer' while less well-educated individuals use 'tumor' when the diagnosis is a cancer related disease. Data on demographic characteristics and clinical information including age, gender, educational status, cancer site and time since diagnosis were extracted from case records. All participants in the study were gastrointestinal cancer patients who were diagnosed during one year ago. Patients who had cognitive problems or were too sick to participate in the interview were excluded.

### Instruments

Quality of life was assessed using the European Organization for Research and Treatment of Cancer Quality of Life Questionnaire (EORTC QLQ-C30). This is a core cancer-specific questionnaire containing 30 items on patient's functioning, global quality of life, disease- and treatment related symptoms [12]. It incorporates five functional scales (physical, role, cognitive, emotional, and social), three symptom scales (fatigue, pain and nausea and vomiting), and a global health and quality of life scale. The remaining single items assess additional symptoms commonly reported by cancer patients (dyspnoea, appetite loss, sleep disturbance, constipation, and diarrhea) and also the perceived financial impact of the disease and treatment. With the exception of two questions concerning physical condition and global quality of life that were rated on a 7-point scale, responses to all other questions were on a 4-point scale (not at all, a little, quite a bit, very much). The psychometric properties of the Iranian version of the EORT QLQ-C30 are well documented. The reliability coefficient for multi-item scales reported to range from 0.48 to 0.98. Validity (performing known-groups comparison analysis), also showed that all the functional and symptom scales discriminated between subgroups of patients differing in clinical status as defined by their performance status and disease stage [13].

### Statistical analysis

In accordance with procedures recommended by the EORTC, score were linearly converted to a scale ranging from 0 and 100 for each patient. For the functional and global quality of life scales, higher scores represent a better level of functioning. For the symptoms scales, higher scores represent worse conditions. The independent samples t-test was used for group comparisons. A *P*-value of less than 0.05 was considered as statistically significant. Then, if the differences between two groups were significant analysis of variance was performed while knowledge of diagnosis, educational status and cancer site considered as fixed factors and age as covariate in order to see whether the factors and covariate had any independent effects on differences observed.

### Ethics

Verbal consents obtained from all patients prior to interview. With regard to cancer disclosure it is worth noting that the information was being withheld from the patient regardless of the present study. In Iran usually physicians or relatives do not disclose cancer diagnosis to patients, although at present there are some changes to the usual practice. In addition, all patients get treatments they needed or would have normally received. Patients were asked to participate in the study to measure their health-related quality of life while indicating we are using this information for our research purposes. The study received two approvals: one from Shahed University and one from Cancer Institute, affiliated to Tehran University of Medical Sciences.

## Results

### Patients' characteristics

In all 165 patients were approached during the recruitment period. Of these 15 patients were excluded due to exclusion criteria and 8 patients refused to participate in the study due to dislike. Finally 142 patients were entered into the study, giving 86% participation rate. The mean age of patients was 54.1 (SD = 14.8) years, most patients were married (86%), male (56%), illiterate (55%). Only 48% knew their cancer diagnosis whereas 52% did not know. The diagnosis was as fallows: stomach (30%), esophagus (29%), colon (22%), rectum (16%), and small intestine (3%). The patients' demographic and clinical characteristics are shown in Table 1.

**Table 1:** Patients' demographic and clinical characteristics

	All (n = 142)	Did not know diagnosis (n = 74)	Knew diagnosis (n = 68)	P
	**No (%).**	**No. (%)**	**No. (%)**	

**Age**				0.001

Mean (SD)	54.1 (14.8)	58.2 (13.4)	50.2 (13.9)	

Range	19–76	19–76	23–74	

**Gender**				0.19

Male	79 (56)	45 (61)	34 (50)	

Female	63 (44)	29 (39)	34 (50)	

Marital status*				0.71

Single	13 (9)	6 (8.1)	7 (10.3)	

Married	122 (86)	63 (85.1)	59 (86.8)	

Widowed	7 95)	5 (6.8)	2 (2.9)	

Educational status*				0.001

Illiterate	78 (55)	55 (74.3)	23 (33.8)	

Primary	43 (30)	15 (20.3)	28 (41.2)	

Secondary	12 (8.5)	3 (4.1)	9 (13.2)	

College/university	9 (6.5)	1 (1.4)	8 (11.8)	

**Cancer site**				< 0.001

Esophagus	41 (29)	34 (45.9)	7 (10.3)	

Stomach	42 (30)	20 (27)	22 (32.4	

Small intestine	5 (3)	4 (5.4)	1 (1.5)	

Colon	31 (22)	5 (6.8)	26 (38.2)	

Rectum	23 (16)	11 (14.9)	12 (17.6)	

**Time since diagnosis**				

Mean (SD)	4.4 (3.2)	4.1 (3.2)	4.6 (3.0)	0.31

Range	1–12	1–12	1–12	

* To carry out a valid test, the cells were merged.

### Quality of life

Comparison of functioning and global quality of life scores between those knew their diagnosis and those who did not it was found that those who knew their diagnosis showed a significant lower degree of physical (P = 0.001), emotional (P = 0.014) and social functioning (P < 0.001). Also the findings indicated that there were no statistically significant differences between two groups in symptoms scores, except for fatigue (P = 0.014) and financial difficulties (P = 0.005). The results are shown in Table 2.

**Table 2:** Patients' quality of life scores as measured by the EORTC QLQ-C30

	Did not know diagnosis (n = 74)	Knew diagnosis (n = 68)	
	**Mean (SD)**	**Mean (SD)**	**P**

Functioning*			

Physical functioning	84.7 (15.9)	74.7 (19.4)	0.001

Role functioning	67.3 (25.9)	69.8 (29.1)	0.58

Emotional functioning	69.4 (20.8)	60.2 (23)	0.014

Cognitive functioning	97.0 (10)	93.2 (16.3)	0.09

Social functioning	88.3 (16.2)	75.5 (24.2)	< 0.0001

Global health, quality of life	65.0 (18.7)	59.5 (25.2)	0.14

Symptoms**			

Insomnia	29.3 (32.6)	35.8 (36.1)	0.26

Fatigue	25.2 (19.3)	34.5 (24.8)	0.014

Pain	32.6 (27.3)	35.8 (31.8)	0.53

Dyspnoae	6.3 (14.2)	7.3 (20.6)	0.72

Constipation	13.5 (29.1)	21.4 (33.7)	0.14

Appetite loss	32.4 (32.5)	40.3 (36.9)	0.18

Diarrhoea	4.9 (15.3)	7.8 (16.4)	0.28

Nausea and vomiting	20.5 (29.4)	17.4 (25.8)	0.5

Financial difficulties	40.0 (37.8)	58.3 (38.4)	0.005

* The higher values indicated a higher level of functioning and quality of life, min.: 0, max.: 100

** The higher values indicate a greater degree of symptoms, min.: 0, max.: 100

Since there were significant differences in age, educational status and cancer site between two groups, further analysis was carried out to examine whether these had any effects on physical, emotional, and social functioning, fatigue and financial difficulties. The results are shown in Table 3. There were no clear patterns for effects of independent variables studied on outcomes that are physical, emotional and social functioning or fatigue and financial difficulties, but in all instances knowledge of diagnosis showed significant effects on observed differences between two groups.

**Table 3:** The results obtained from analysis of variance while functioning and symptom scores considered as dependent variables and knowledge of cancer diagnosis, educational status and cancer site considered as fixed factors and age as a covariate.

Outcomes	Covariate and fixed factors	F statistics	P
*Physical functioning*			

	Age	0.55	0.45

	Educational status	1.23	0.29

	Cancer site	2.14	0.07

	Knowledge of diagnosis	6.73	0.01

*Emotional functioning*			

	Age	6.61	0.01

	Educational status	2.31	0.08

	Cancer site	1.51	0.20

	Knowledge of diagnosis	5.05	0.02

*Social functioning*			

	Age	0.079	0.78

	Educational status	3.01	0.03

	Cancer site	2.57	0.04

	Knowledge of diagnosis	4.71	0.03

*Fatigue*			

	Age	0.180	0.67

	Educational status	0.698	0.55

	Cancer site	2.45	0.05

	Knowledge of diagnosis	4.67	0.03

*Financial difficulties*			

	Age	0.096	0.75

	Educational status	0.918	0.43

	Cancer site	1.28	0.25

	Knowledge of diagnosis	5.45	< 0.0001

## Discussion

This was a study that compared quality of life in gastrointestinal cancer patients who knew their cancer diagnosis and who did not. The most striking findings of the study were the fact that patients who did not know their cancer diagnosis showed better conditions on a number of quality of life subscales as measured by the EORTC QLQ-C-30. The findings indicated that those who knew their diagnosis showed a significant lower degree of physical, social and emotional functioning. A study of lung cancer patients also showed comparable results [14]. Similarly in Turkey and India it has been demonstrated that psychiatric disorders occurred to lesser extent in patients who were not aware of their cancer diagnosis. The authors concluded that these patients had a more hopeful outlook to the outcome of treatment [8,15]. However, studies from different cultural backgrounds suggested that awareness of cancer diagnosis and prognosis does not itself cause emotional distress and honest disclosure of truth does not worsen any dimension of quality of life [9,16,17].

In Iran the majority of physicians do not inform caner patients about their true nature of illness, and most patients who know their diagnosis obtain information indirectly [18] and thus this might lead to a higher level of emotional distress in patients who become aware of their illness [19]. Studies from Greece also indicated that although physicians have the tendency to tell the truth more often today than in the past, the majorities still disclose the truth to the next of kin [20]. However, this hypothesis warrants further validation.

There were no differences between symptoms scales among patients who knew the cancer diagnosis and who did not, except for fatigue that was higher in aware patients. The high level of fatigue in this group could not be due to age of patients, because the unaware patients were younger. It is demonstrated that fatigue is more frequent in elders [21], and in cancer patients age and fatigue are not closely related [22]. One might argue since emotional distress was higher in patients who knew the diagnosis thus they were more fatigued [23]. Our own findings indicated that patients who scored higher on emotional functioning were less fatigued while those who scored lower on emotional functioning scored higher on fatigue scale (Table 2).

It is difficult to explain the superior physical functioning in unaware patients. However, the finding might be explained by the fact that the questions regarding physical functioning (items 1 to five from the EORTC QLQ-C30) were more global and most patients who knew their diagnosis indicated that they did not want to have a walk or that they were too worried and preferred to stay at home. For instance, when these patients were asked 'do you have any problem in taking a long walk?'; thirty-seven out of 68 replied quite a bit and very much. Thus, the physical functioning in this group was lower than comparison group.

There appear to be a strong relationship between illiteracy and not knowing the diagnosis. The relevance of level of education and knowledge of cancer diagnosis seems worthwhile to be examined in the future studies. As we suggested, with regard to cultural and resources of medical services in the Middle East there should be two different strategies for disclosure of cancer diagnosis: one for illiterate or less educated people and one for people with higher education [24]. However, evidence indicates that sensible disclosure of diagnosis and prognosis is important and satisfaction with information-giving is associated with a better quality of life [25]. Unfortunately we did not study this and to come to a sensible conclusion about knowledge of cancer diagnosis and its effects on quality of life in the future studies it is necessary that the attitudes of physicians about cancer disclosure also be examined. In other words one might argue that it is not the knowledge of cancer diagnosis that affects quality of life and rather it is the physicians' information-giving manner that contributes to such findings.

With regard to disclosure of cancer diagnosis three initial options could be assumed: physicians do not believe in disclosing bad news to cancer patients; patients do not want know their cancer diagnosis; and finally family caregivers do not allow physicians to disclose the diagnosis to cancer patients. An interesting population-based study from Sweden studying 907 widowers whose wives had died of cancer revealed that although a large majority of men prefer an immediate disclosure about the incurable stage of their wife's illness, 41% of the husbands received this information during the last week of the patient's life or not at all [26]. As far as we know for instance in Iran it is estimated that about 80% of physicians do not disclose the cancer diagnosis to patients, although most patients want to know the true diagnosis [18]. Similarly a study from Taiwan showed that the arguments that cancer patients from an Asian culture (that is Chinese/Taiwanese culture) have different preferences regarding being informed of their diagnosis and prognosis and that family member have legitimate right in decision making could not be supported by data obtained from terminally-ill cancer patients [27]. As suggested in many traditional cultures (e.g. most Asian countries or a few European cultures) an effort to protect patient from despair and feeling of hopelessness, family caregivers more often exclude patients from the process of information exchange. Thus, in such societies family plays an important role in the provision of care and information disclosure and they usually decide on the patients' behalf [28]. However, the effect of such protections on patients' quality of life is unclear. An investigation from Taiwan, studying 1108 dyads of patient-family caregivers showed that patient awareness of prognosis, patient-family caregivers agreement on the preferred place of death, and the subjective family caregivers burden had a significant effect on the quality of life of terminally ill cancer patients [29].

This study showed that, unlike reports from western countries, there was a lower awareness of cancer diagnosis in our study group. In general in most developing countries physician-patient communication is poor. The other main reason for not informing patients in Iran is that most people, as in many middle-eastern countries, interpret the diagnosis of cancer as equivalent to death and therefore families may request physicians not to tell the patient the truth [18].

Finally, one should be cautious that this study suffers from some limitations. As suggested the argument that cancer patients from an Asian culture have different preferences about being informed of their diagnosis and prognosis that dictate significantly modifying information disclosure practices should be guided by direct investigation of attitudes regarding information disclosure from cancer patients in Asian cultures and the impact of knowing diagnostic information on the outcomes (such as QOL) of cancer patients [27,29]. Furthermore, at the time of current project the Iranian version of the EORTC QLQ-STO22, a site-specific measure of quality of life in patients with gastric cancer was not available to use in this study. Therefore it is important that in the future studies to supplement the QLQ-C30 with the QLQ-STO22 [30] to have a better understanding on the topic.

## Conclusion

In conclusion, the study findings showed that patients who did not know their cancer diagnosis had a better physical, social and emotional quality of life. It seems that due to cultural differences between countries cancer disclosure guidelines perhaps should be differing.

## Competing interests

The authors declare that they have no competing interests.

## Authors' contributions

AT was the main investigator and wrote the first draft of the manuscript. MAM contributed to patient recruitments. RR supervised the study. ZT contributed to the data collection. AM supervised the study, analyzed the data and wrote the final draft of the manuscript. All authors read and approved the final manuscript.

## Pre-publication history

The pre-publication history for this paper can be accessed here:

http://www.biomedcentral.com/1471-2407/9/39/prepub
